# Unraveling the potential of urban greenery for extreme heat and ozone mitigation in Northern Hemisphere cities

**DOI:** 10.1093/nsr/nwag494

**Published:** 2026-08-17

**Authors:** Mengmeng Li, Yuting Lu, Xin Huang, Hui Zhang, Zilin Wang, Yue Qin, Jun Ge, Xueyu Zhou, Wei Wang, Wenjie Wang, Nan Wang, Tijian Wang, Minghuai Wang, Haishan Chen, Aijun Ding

**Affiliations:** State Key Laboratory of Severe Weather Meteorological Science and Technology, Nanjing University, Nanjing 210023, China; School of Atmospheric Sciences, Nanjing University, Nanjing 210023, China; Frontiers Science Center for Critical Earth Material Cycling, Nanjing 210023, China; School of Atmospheric Sciences, Nanjing University, Nanjing 210023, China; State Key Laboratory of Severe Weather Meteorological Science and Technology, Nanjing University, Nanjing 210023, China; School of Atmospheric Sciences, Nanjing University, Nanjing 210023, China; Frontiers Science Center for Critical Earth Material Cycling, Nanjing 210023, China; School of Atmospheric Sciences, Nanjing University, Nanjing 210023, China; State Key Laboratory of Severe Weather Meteorological Science and Technology, Nanjing University, Nanjing 210023, China; School of Atmospheric Sciences, Nanjing University, Nanjing 210023, China; College of Environmental Sciences and Engineering, Peking University, Beijing 100871, China; School of Atmospheric Sciences, Nanjing University, Nanjing 210023, China; School of Atmospheric Sciences, Nanjing University, Nanjing 210023, China; Nanjing-Helsinki Institute in Atmospheric and Earth System Sciences, Nanjing University, Suzhou 215163, China; Nanjing-Helsinki Institute in Atmospheric and Earth System Sciences, Nanjing University, Suzhou 215163, China; College of Carbon Neutrality Future Technology, Sichuan University, Chengdu 610207, China; School of Atmospheric Sciences, Nanjing University, Nanjing 210023, China; State Key Laboratory of Severe Weather Meteorological Science and Technology, Nanjing University, Nanjing 210023, China; School of Atmospheric Sciences, Nanjing University, Nanjing 210023, China; Frontiers Science Center for Critical Earth Material Cycling, Nanjing 210023, China; State Key Laboratory of Climate System Prediction and Risk Management, Nanjing University of Information Science & Technology, Nanjing 210044, China; School of Atmospheric Sciences, Nanjing University, Nanjing 210023, China; Nanjing-Helsinki Institute in Atmospheric and Earth System Sciences, Nanjing University, Suzhou 215163, China

**Keywords:** urban greenery, cooling, extreme heat, ozone, Northern Hemisphere cities

## Abstract

Escalating exposure to extreme heat and ozone (O_3_) pollution has emerged as a principal challenge for cities worldwide. While urban greening is widely advocated for mitigating extreme heat, its impacts on compound heat-O_3_ pollution remain poorly understood due to intricate urban meteorology-chemistry coupling. By integrating observations, reanalysis data, and urban-land-atmosphere modeling, we demonstrate that urban greenery provides a notable yet uneven cooling effect of −0.26°C to −1.08°C in Northern Hemisphere’s metropolises, shaped by greenery fraction and local climate. Furthermore, the nonlinearly weakened O_3_ photochemical production within a greenery-stabilized urban boundary layer is capable of decreasing O_3_ in populated city centers. Despite the dual positive effects in alleviating heat and O_3_ exposures meteorologically, greenery-boosted biogenic emissions could chemically raise urban O_3_ via supplementing precursors, totally by up to 7.6 µg m^−3^. These findings underscore the need to incorporate fully coupled urban meteorology-chemistry interactions in urban greening strategies to achieve climatic and environmental co-benefits.

## Teaser

Though urban greenery alleviates heat and O_3_ stress meteorologically, greenery-boosted biogenic emissions could chemically fuel O_3_ formation via supplementing precursors.

## INTRODUCTION

Rapid economic growth has led to widespread urban sprawl globally, with more than half of the world’s population residing in cities, and the urban population projected to reach 70% by 2050 [1]. Urban overheating, driven by global warming and local urban effects, has led to escalating heat exposures within cities [2] and emerging challenges to urban sustainability, such as increased mortality and morbidity [3], a surge in energy demand [4], and heat-related societal inequity [5]. Moreover, ozone (O_3_) pollution, a major air quality concern in urban environments, tends to deteriorate sharply during heat extremes [6,7]. The concurrent occurrence of extreme heat and elevated O_3_ levels therefore poses a severe threat to public health and has become one of the most critical hazards facing cities.

Large-scale greening has been proposed as a nature-based solution to mitigate climate warming, by functioning as efficient carbon sinks [8], and as a modifier of surface energy balance through enhanced evapotranspiration or shading of incident solar radiation [9,10]. Recent studies have revealed a notable greening trend in 41% of urban areas worldwide due to municipal greening initiatives [11,12], as well as elevated CO_2_ and temperature-driven fertilization effects under global warming [13]. Notably, urban green spaces, such as parks, gardens, greenways, and street trees, play a pivotal role in mitigating urban heat but exhibit considerable spatial disparities [5,14,15]. For example, the Global North cities exhibit higher greening ratios and stronger cooling effects compared to those in the Global South (3.6 ± 1.7°C and 2.5 ± 1.0°C, respectively, for land surface temperature) [5]. This inequality in cooling adaptation is closely related to the quantity and quality of urban green spaces, shaped by local climate [15], urban morphology [16], and tree traits [17]. Quantifying the cooling potentials of urban greenery at hemispheric or global scales remains challenging due to the pronounced climatic variabilities and heterogeneous configurations of green spaces across cities.

Moreover, although urban greening is widely promoted as a cost-effective strategy to mitigate extreme heat, its associated effects on compound heat-O_3_ pollution events remain inadequately understood, particularly given diverse urban climate as well as their intricate coupling with atmospheric chemistry [18]. Recent research has indicated that afforestation in South China alleviated O_3_ pollution via enhancing dry deposition and suppressing turbulence mixing [19]. In contrast, other studies suggested that greening may exacerbate urban O_3_ pollution due to deteriorated diffusion conditions and altered wind dynamics [20,21]. Meanwhile, despite the relatively low densities of urban flora, emissions of biogenic volatile organic compounds (BVOCs) can be considerable because of the higher ambient temperatures and widespread planting of high-emitting trees in cities [22,23]. For instance, urban BVOCs account for 15%–20% of total volatile organic compound (VOC) emissions in Chinese cities [24], and heatwaves can trigger BVOC emission surges from urban herbaceous plants [25]. Growing evidence has demonstrated that BVOC emissions significantly exacerbated O_3_ pollution in VOC-limited urban environments [26,27], further complicating the role of urban greenery in compound heat-O_3_ pollution events. However, the extent to which urban greenery modulates these compound hazards remains poorly investigated, especially considering the intricate interactions among vegetation, urban meteorology, and atmospheric chemistry.

In the context of on-going global warming, there has been a rise in the simultaneous occurrence of extreme heat and elevated O_3_ levels, particularly in the densely populated and rapidly urbanizing areas of the Northern Hemisphere [7]. Understanding the potential of urban greenery to mitigate compound heat-O_3_ events is therefore essential for informed urban planning and sustainable city development. In the present study, by integrating ground-based observations, reanalysis products, and urban-land-atmosphere modeling with realistic representations of urban form and greenery, we provide a comprehensive assessment of how urban vegetation modulates compound heat-O_3_ hazards and associated population exposures in major metropolises of the Northern Hemisphere.

## RESULTS

### Escalating compound heat-ozone extremes in Northern Hemisphere cities

The Northern Hemisphere has experienced its warmest decade of the past century (Fig. 1a), characterized by a positive surface air temperature anomaly approaching 1.5°C above the climatological averages, attributed in part to anthropogenic greenhouse gas emissions and land cover changes [2]. As global temperatures continue to rise, Northern Hemisphere cities have repeatedly recorded unprecedented summer heat in recent years. Long-term analysis clearly demonstrates an intensification of summertime heat extremes, defined as days when daily maximum air temperature (*T*_max_) surpasses the threshold of 32°C (see Methods), which is the World Meteorological Organization (WMO)’s threshold for a heat-stress alert (Fig. 1b). Both the frequency and severity of heat extremes have increased markedly from 1980 to 2023: their occurrence has risen at 1.0 days decade^−1^, while their intensity has escalated at 6.6°C decade^−1^ (*P* < 0.05). These trends underscore the accelerating emergence of dangerous heat conditions across the Northern Hemisphere. Notably, there is no universally accepted definition of heat extreme; for example, China Meteorological Administration suggests a fixed *T*_max_ threshold of 35°C, while many studies identify heat extremes using percentile-based thresholds, such as the 90th percentile of *T*_max_ distribution. The spatial patterns of summer heat extremes derived from fixed-temperature thresholds are generally consistent, despite quantitative differences, with high occurrences concentrated in the mid-low latitudes and arid zones of the Northern Hemisphere (Fig. S1). Comparatively, percentile-based thresholds yield a more spatially uniform distribution (Fig. S1). Since the interactions between temperature and O_3_ concentration depend on absolute temperature magnitude, we adopt WMO’s fixed-temperature threshold for analysis.

**Figure 1.**
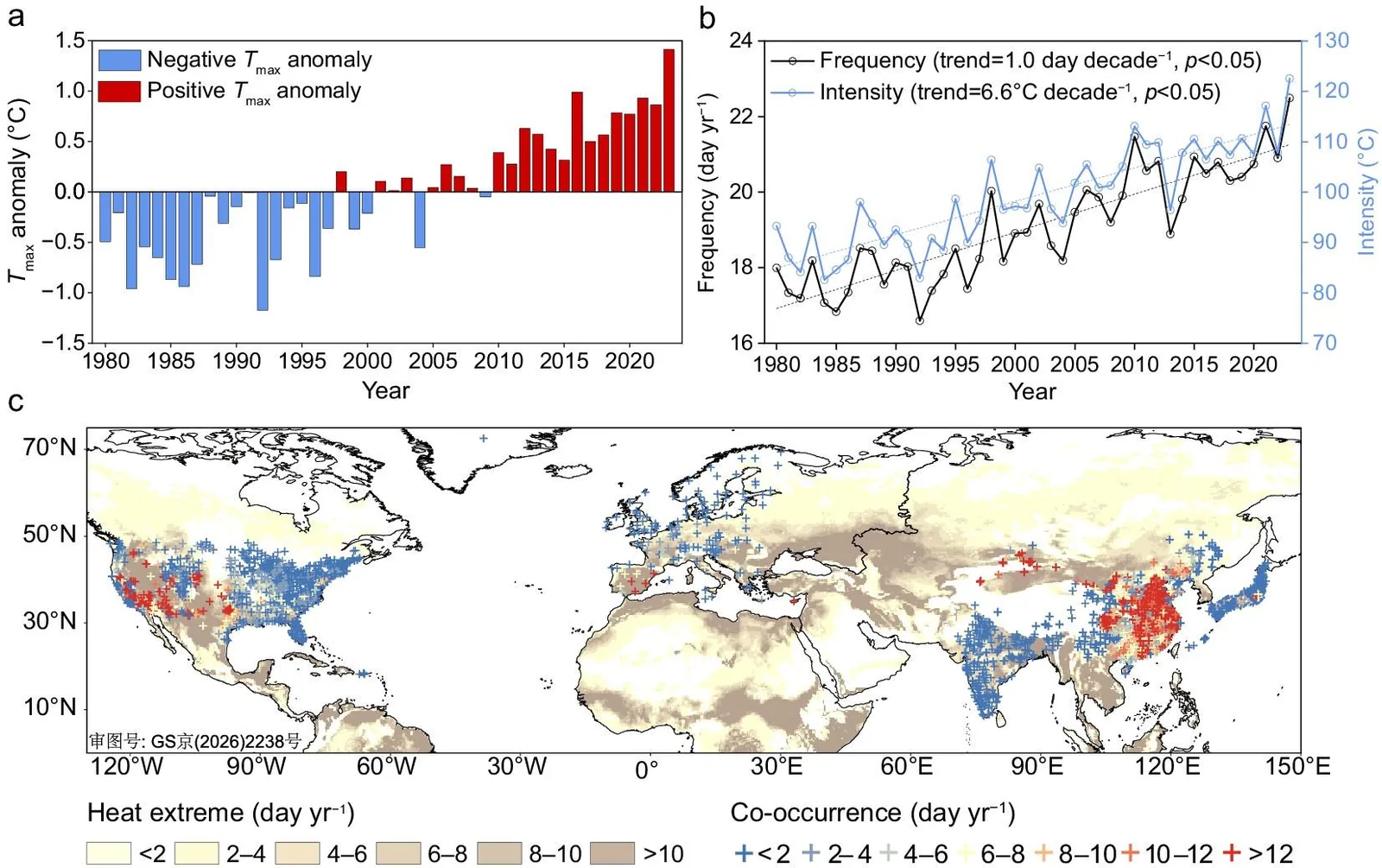
Increased co-occurrence of heat and O_3_ extremes across the Northern Hemisphere under a warming climate. (a) Summer *T*_max_ anomalies relative to the climatological averages in the Northern Hemisphere since 1980. (b) Increasing frequency and intensity of heat extremes in the Northern Hemisphere since 1980. (c) Annual frequency of compound heat-O_3_ extremes (cross symbols) between 2014 and 2023. Shaded areas denote the increased heat extremes between 2014 and 2023 relative to the 1980s.

At the same time, rapid urbanization has intensified human activities and driven substantial emissions of air pollutants, leading to a marked deterioration in urban air quality. Using June–August surface O_3_ observations from major Northern Hemisphere countries over the past decade (2014–2023; see Methods), we reveal that O_3_ pollution has emerged as a pervasive environmental challenge in the Northern Hemisphere, particularly in the North China Plain (NCP), the Yangtze River Delta (YRD), and the Sichuan Basin in China, as well as the Southwest and Intermountain West regions of the USA, and the alpine areas of Central Europe (Fig. S2a). In these regions, summer maximum daily 8-h average (MDA8) O_3_ concentrations have consistently exceeded the World Health Organization’s Interim Target II of 120 μg m^−3^, attributable to intensive precursor emissions and active photochemical reactions. Notably, the deseasonalized and detrended MDA8 O_3_ concentrations show a positive correlation with *T*_max_, with the correlation coefficients (*R*) exceeding 0.70 (Fig. S2b). During heat extremes, the exceptionally high air temperatures substantially elevate surface O_3_ concentrations by 13.21 μg m^−3^ (14.30%), 29.79 μg m^−3^ (34.11%), and 27.69 μg m^−3^ (25.61%), respectively, in the USA, Europe, and China compared to normal days (Fig. S2c). Prolonged high air temperatures and elevated O_3_ frequently co-occur for more than 10 days per year (defined as the total number of days when both *T*_max_ exceeds 32°C and MDA8 O_3_ exceeds 120 μg m^−3^) in the NCP and YRD regions of China, as well as southern California and mid-eastern USA (Fig. 1c). Notably, the frequency of compound heat-O_3_ events defined using different temperature and O_3_ thresholds varies in magnitude but exhibits consistent spatial patterns (Fig. S3), with hotspots concentrated in the densely populated and highly polluted urban agglomerations (UA). Such simultaneous occurrence of extreme heat and O_3_ pollution represents a substantial risk to public health and has emerged as a primary urban environmental hazard in Northern Hemisphere cities.

Particularly, during the unprecedented hot summer of 2022, most midlatitudes in the Northern Hemisphere experienced exceptionally high air temperatures accompanied by pronounced positive O_3_ anomalies, particularly in the USA and Canada, western Europe, and the lower-middle reaches of Yangtze River in China (Fig. 2a). To elucidate the potential of urban greenery for extreme heat and O_3_ mitigation, we then focus on five metropolitan regions most severely affected by the 2022 heatwaves, that is, the California UA in the southwestern USA, the Atlantic Coast UA in the northeastern USA, the London- and Paris-centered UA in western Europe, the YRD UA in China, and the Tokyo-Pacific Coast UA in Japan (Fig. 2b). These UAs are among the world’s largest economic zones and rapidly developing metropolises. They span temperate marine climate in the London- and Paris-centered UA, and the Tokyo-Pacific Coast UA, to Mediterranean climate in the California UA, temperate continental humid climate in the Atlantic Coast UA, as well as subtropical monsoon climate in the YRD UA. During the summer of 2022, all five UAs experienced anomalously high air temperatures, coinciding with significant positive O_3_ anomalies over 30 μg m^−3^ (Fig. S4). The occurrence frequency of compound heat-O_3_ events during 2022 summer varied widely across regions, ranging from fewer than 5 days in these high-latitude UAs to more than 40 days in major Asian metropolises (Fig. 2b), reflecting great discrepancy in compound heat-O_3_ hazards across diverse climatic and urban settings.

**Figure 2.**
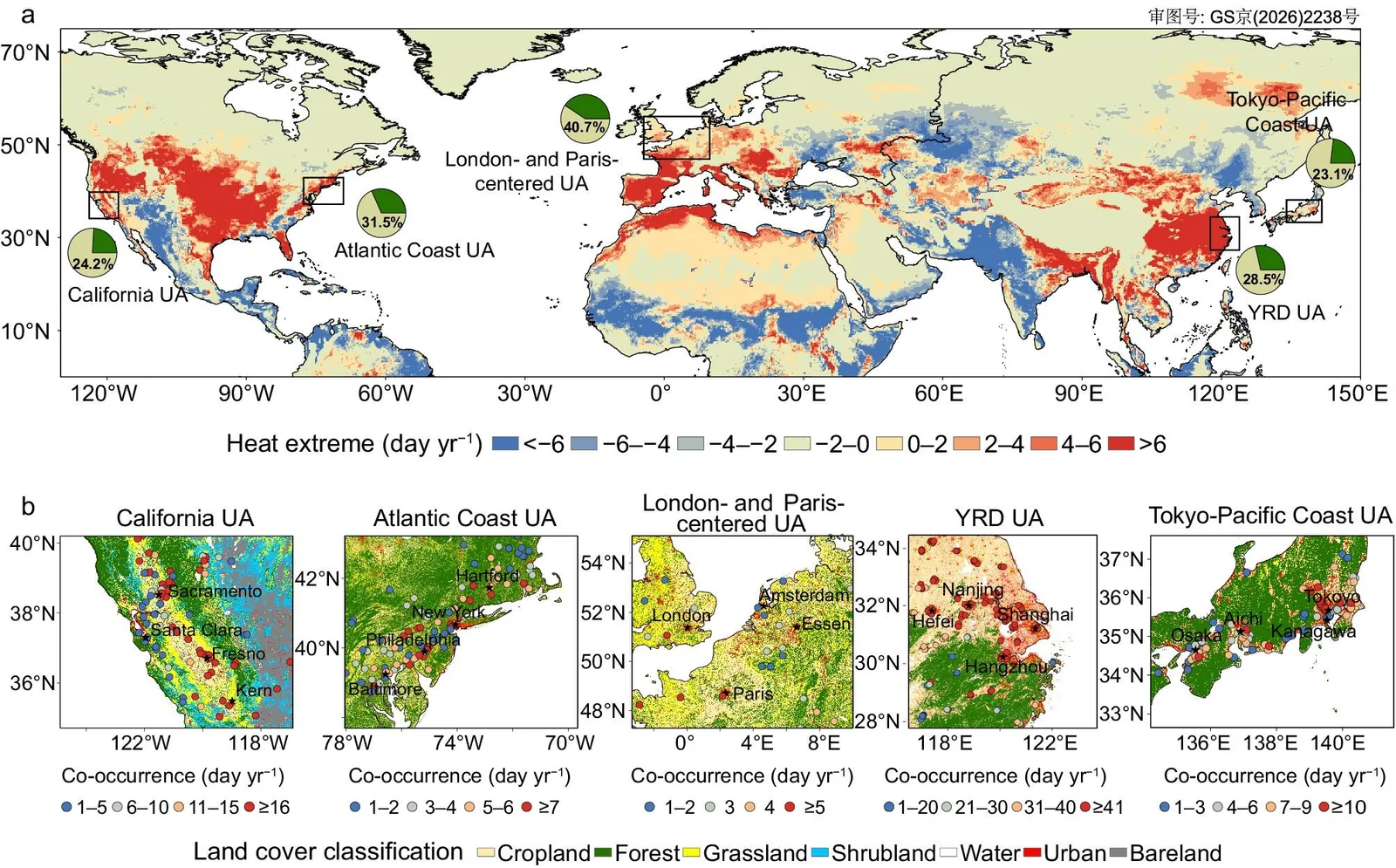
Unprecedented hot summer of 2022 led to increased risks of compound heat-O_3_ extremes in the Northern Hemisphere cities. (a) Elevated frequency of heat extremes during 2022 summer compared to the 2014–2023 averages. Green pies and values denote the proportions of green space in urban centers of the five UA, which is derived from the high-resolution FROM-GLC dataset. (b) Frequency of compound heat-O_3_ extremes (colored dots) during 2022 summer in the five UA. Shaded areas denote the land cover types derived from FROM-GLC dataset. Black stars denote the cities selected for model analysis in Fig. 5.

### Benefits of urban greenery in mitigating urban heat stress

Amid accelerated urbanization, many nations have incorporated green spaces into their urban planning strategies. European Union (EU) has launched the 3 Billion Trees Initiative to promote creation of green corridor and urban forest [28]. Likewise, China has advanced a series of large-scale greening programs, including the National Garden City program launched in 1992, the National Forest City program initiated in 2004, and the National Ecological Garden City program commenced in 2016 [29]. Urban greenness retrieved from satellite-based normalized difference vegetation index (NDVI) shows a substantial increase of 14.70%, 4.06% and 3.69% from 2010 to 2022 in the urban centers (i.e. high-density residential areas and commercial centers within the urban areas; see Methods) of the YRD, Tokyo-Pacific Coast, and Atlantic Coast UAs (Fig. S5a). These increases reflect not only the deliberate human-driven greening activities, but also the fertilization effects associated with elevated CO_2_ and temperatures under global warming [13].

Utilizing the Weather Research and Forecasting (WRF) model, in conjunction with a single-layer urban canopy model (SLUCM) and realistic representation of urban landscape (see Methods), we assess the effectiveness of greenery in mitigating urban heat during the unprecedented hot summer of 2022. The 10-m Finer Resolution Observation and Monitoring of Global Land Cover (FROM-GLC10) dataset [30] is employed to depict the distribution of urban greenery in metropolitan regions (see Methods; Fig. S6). Analysis reveals a substantial yet highly uneven distribution of urban green spaces across the five UAs (Fig. 2a). Urban centers along the Atlantic Coast, and those centered around London and Paris, demonstrate considerable levels of urban greenery, with a high proportion of 31.6% and 40.7%, respectively. In contrast, the densely populated Asian megacities and the California UA, located in an arid environment, show much lower greenery fractions of typically 20%–30%, consistent with the satellite-retrieved NDVI patterns (Fig. S5b). Model evaluation indicates that the WRF-SLUCM model is capable of accurately capturing the near-surface air temperature, achieving a *R* ranging from 0.95 to 0.98, and a negative normalized mean bias (NMB) of −9% to −2% for 2-m air temperature (Fig. S7).

The cooling effect and cooling efficiency of urban greenery, defined as the reduction in 2-m air temperature corresponding to per 100% increase in urban vegetation cover, serve as metrics to assess its influence on urban thermal environments. Urban greenery could mitigate urban heat by modifying surface energy balance through enhanced transpiration or shading of radiation (see Methods) [9,10]. This phenomenon results in a significant reduction of surface sensible heat by −35.43 to −72.18 W m^−2^ in urban areas, while simultaneously increasing latent heat by 39.45–94.32 W m^−2^ (Fig. S8). As a result, a pronounced cooling effect emerges across these metropolitan regions, with a reduction in summer-average *T*_max_ ranging from −0.26°C to −1.08°C (Fig. 3a). This effect is supported by the negative correlation between satellite-derived land surface temperature and urban NDVI (*R* = −0.61 to −0.91, *P* < 0.05) observed in several capital cities (Fig. S9).

**Figure 3.**
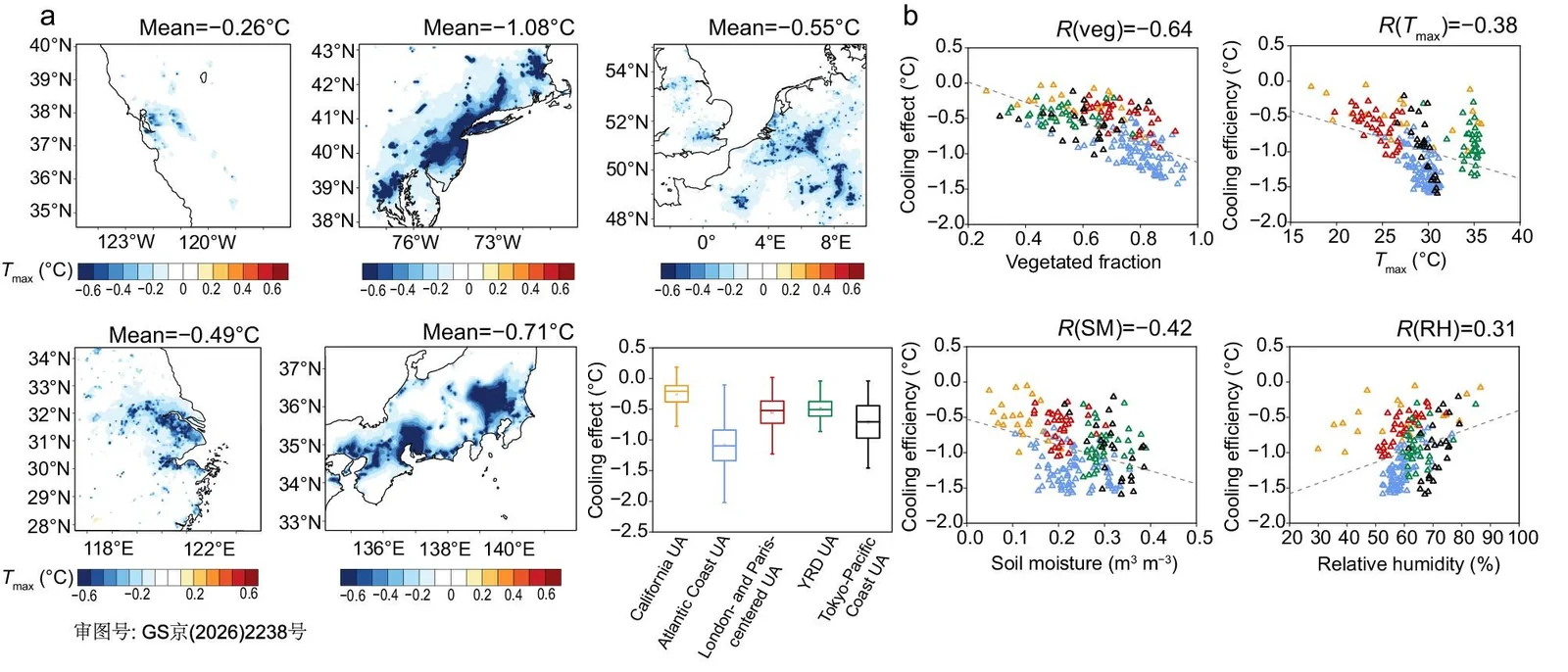
Cooling effect and efficiency of urban greenery in the five metropolitan regions of Northern Hemisphere. (a) Cooling effect of urban greenery in terms of summer-average *T*_max_ in the five metropolitan regions from WRF-SLUCM urban climate simulations. The average values over urban areas are given in panel (a). Box charts denote the 25th and 75th percentiles of cooling effects in the urban areas within each metropolitan region. (b) Dependence of greenery-induced cooling effect and cooling efficiency on the vegetated urban fraction and local climatic conditions in 198 cities, including 21 counties in the California UA, 83 counties in the Atlantic Coast UA, 35 cities in the London- and Paris-centered UA, 34 cities in the YRD, and 25 cities in the Tokyo-Pacific Coast UA. Different colors of triangles denote different metropolitan regions as shown in panel (a). The correlation coefficients (*R*) are given in panel (b).

Notably, the cooling effects of urban greenery vary significantly across regions. The strongest cooling occurs in the Atlantic Coast UA (−1.08 ± 0.34°C), whereas the California UA exhibits markedly weaker cooling (−0.26 ± 0.20°C). City-level cooling from 198 cities further illustrates this pronounced spatial disparity in cooling effects, due to diverse urban vegetation fractions and the region-specific cooling efficiencies shaped by local climatic conditions (Fig. 3b). The significant negative correlation between the cooling effect and vegetated urban fraction (*R* = −0.64, *P* < 0.05) indicates that an increase in urban greenness can strongly mitigate urban heat stress. Additionally, the cooling efficiency of greenery is negatively correlated with soil moisture (SM; *R* = −0.42, *P* < 0.05), which can be ascribed to the enhanced evaporative cooling in moist soil conditions. For instance, peculiarly low cooling efficiency in Californian cities, which are situated in a desert biome, is primarily due to the inherent aridity of local climate. Otherwise, the rising air temperature (*R* = −0.38, *P* < 0.05) and decreasing atmospheric humidity (*R* = 0.31, *P* < 0.05) could elevate vapor pressure deficit, thereby facilitating the heat dissipation and cooling through transpiration. Importantly, greenery-induced cooling tends to double during heatwaves (Fig. S10), as higher air temperatures intensify transpiration and amplify cooling of urban vegetation [31]. We estimate that 12.74–438.46 million people-days relieve from the dampened heat extremes by urban greenery in these metropolitan regions during the unprecedented hot summer of 2022, underscoring it as an effective nature-based solution for extreme heat mitigation.

Notably, the thermal effect of urban vegetation is determined by the balance between albedo-induced radiative warming and evaporative cooling, which does not always lead to cooling, especially under arid or water-limited conditions. Recent observations indicated a rise in daytime surface temperature with increased tree coverage in some African and European cities [32,33]. Guo *et al.* [34] further demonstrated that urban vegetation’s cooling effects weaken in global arid zones, which may even shift from cooling to warming as aridity increases. In humid environments, vegetation generally cools near-surface air through shading and transpiration. However, in arid regions, insufficient soil moisture can suppress transpiration cooling. Under such conditions, grasslands and croplands, which usually have shallow roots and limited access to deep soil water, may lose much of their evaporative cooling capacity. Meanwhile, their lower albedo relative to bare soil or bright surface can increase absorbed radiation, causing weak cooling or even local warming. The WRF/SLUCM is capable of explicitly quantifying the effects of vegetation transpiration on water vapor exchange and latent heat partitioning over urban areas (see Equations S1 and S2). As shown in Fig. S8, the model reproduces the disparities in latent heat fluxes across humid and arid urban environments. Transpiration and latent heat fluxes of vegetation in Californian cities located within desert biomes are markedly lower, with a mean value of 39.45 W m^−2^, compared with other metropolitan areas (68.15–94.32 W m^−2^). Consequently, urban vegetation in Californian UA delivers a far weaker cooling effect (−0.26 ± 0.20°C).

### Urban O_3_ responses to greenery considering meteorology–chemistry coupling

Despite as an effective cooling way for urban heat, urban afforestation’s impacts on O_3_ pollution, an emerging environmental stress accompanied with high temperatures, are still not well understood and quantified in Northern Hemisphere cities. As a secondary pollution formed by photochemical reactions, O_3_ is modulated nonlinearly by precursor emissions, urban meteorology, and their intricate interactions. To quantitatively understand urban greenery’s role in O_3_ pollution and disentangle these complex physicochemical processes, we conducted a set of WRF/Chem-SLUCM urban climate-chemistry coupled simulations in these UAs during typical prolonged heat-O_3_ pollution events (see Methods). The model reproduces the spatiotemporal patterns of O_3_ across UAs (Fig. S11), although it generally overestimates the magnitudes with NMBs ranging from 10% to 34%. This overestimation of summertime O_3_ concentrations may arise from uncertainties in emission inventories and meteorological forcing data [27,35], as well as the overprediction of photolysis rates and radical concentrations in photochemical mechanisms in WRF/Chem [36,37]. Additionally, this study utilizes *in-situ* observations of isoprene concentrations at four representative urban/suburban sites across these UAs (see Methods) to evaluate the model performance of isoprene emission (Fig. S12). Overall, the model reasonably reproduces the diurnal and day-to-day variations of isoprene at four observational sites. However, biases exist in the peak magnitudes: extreme peak values are underestimated at Los Angeles, while daytime peaks are overpredicted to varying degrees at Baltimore, Chilbolton, and Nanjing.

Simulation results reveal that although urban greenery effectively alleviates urban heat, its influence on O_3_ pollution is complex and spatially heterogenous. The substantial cooling induced by urban greenery could suppress atmospheric turbulence within urban boundary layer (UBL), leading to a decrease in daytime UBL height by 23.38–211.52 m on average (Fig. S13). The deteriorated atmospheric diffusion conditions hinder dispersion of air pollutants as well as O_3_ precursors, resulting in an elevated daytime nitrogen dioxide (NO_2_) concentration by 0.13–1.88 μg m^−3^ in urban areas. Due to the nonlinearity of O_3_ photochemistry, urban O_3_ pollution responds differently among regions to urban greenery, influenced by prevailing chemical regimes. The ratio of hydrogen peroxide to nitric acid (H_2_O_2_/HNO_3_), an indicator of O_3_ chemical sensitivity, reveals a pronounced contrast between urban and suburban environments (Fig. 4a). In densely polluted urban centers, O_3_ formation is primarily VOC-limited, defined by a H_2_O_2_/HNO_3_ value below 0.4∼0.5 as proposed by Sillman [38], whereas the surrounding suburban areas tend to fall within NOx-limited or transition regimes. This progressive shift of O_3_ chemical sensitivity is especially prominent in those emission-intensive regions such as the YRD and Tokyo-Pacific Coast UAs (Fig. S14). As a result of greenery-stabilized UBL, the daytime O_3_ photochemical production rate and associated pollution exposure decreases in the urban centers of these metropolitan regions (Fig. S15a), due primarily to surface cooling and elevated NOx levels within a VOC-limited urban environment (Fig. 4a). Conversely, in suburban region, the photochemical O_3_ production is less affected or even enhanced by greenery-modified urban climate (Fig. 4a). Additionally, greenery-induced cooling reduces temperature disparities between urban and suburban regions, which in turn weakens the low-level urban wind convergence (as evidenced in Fig. S16c). This further disrupts the urban heat island-driven inflow that transports O_3_-rich air masses into city centers, whereby high O_3_ preferentially accumulates in suburbs, further amplifying the urban/suburban disparities in O_3_ response. Noteworthy is that the opposite responses of O_3_ to greenery tend to be more pronounced in Asian cities; otherwise, O_3_ pollution is severely aggravated across US cities in both urban centers and suburbs featuring NOx-limited or mixed regimes (Fig. S14).

**Figure 4.**
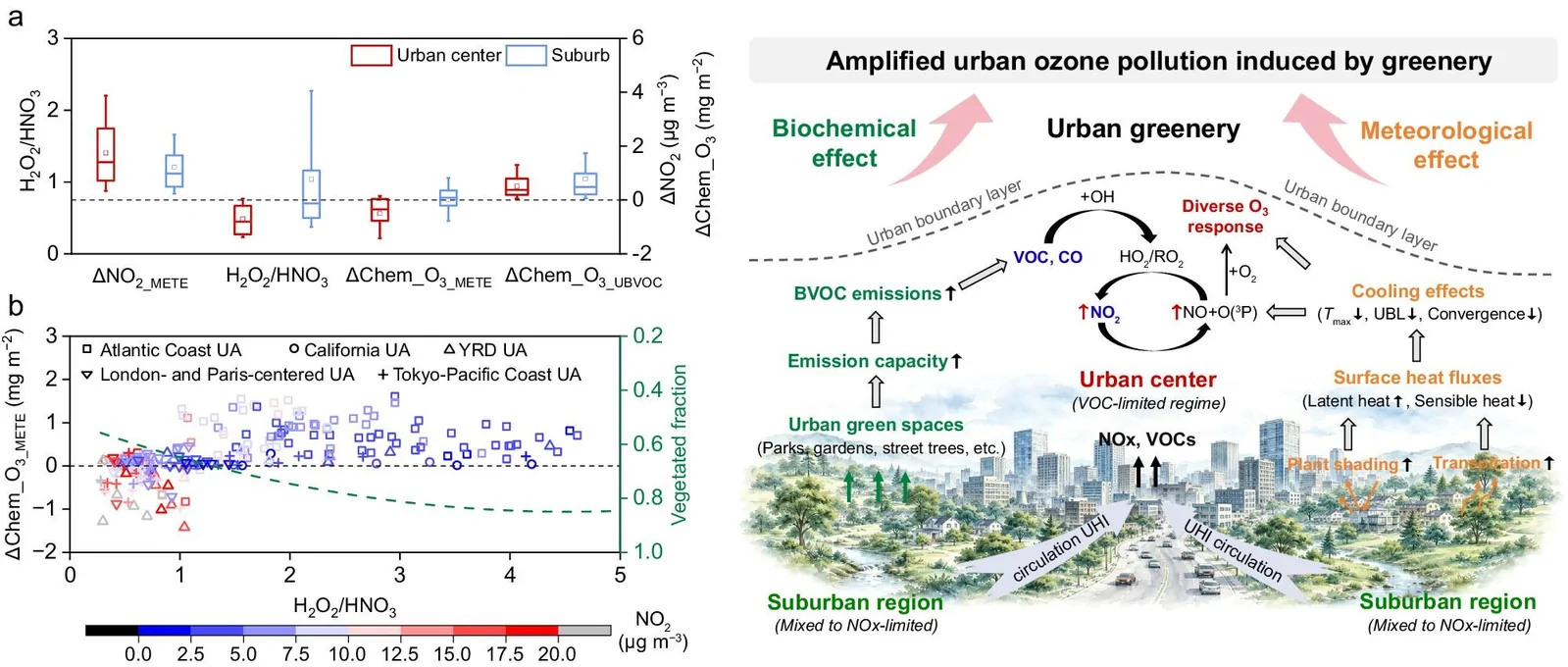
Meteorology–chemistry coupling mechanisms underlying the O_3_ responses to urban greenery. (a) Diverse responses of daytime O_3_ chemical production in urban centers and suburban regions due to greenery-modified urban climates (ΔChem_O_3_METE_) and urban BVOC emissions (ΔChem_O_3_UBVOC_), respectively. ΔNO_2_METE_ denotes the changes in daytime surface NO_2_ concentrations. (b) Changes in daytime O_3_ chemical production due to greenery-modified urban climates depending on H_2_O_2_/HNO_3_ ratio and NO_2_ concentrations (border color) in the urban areas of 198 cities. Green line denotes the relationship between vegetated urban fraction and H_2_O_2_/HNO_3_ ratio. (c) Schematic diagram of the potential mechanism of how urban greenery affects urban O_3_ pollution through meteorological and biochemical pathways. Up and down arrows indicate increase and decrease in a variable.

In addition to modifying urban climate, urban greenery is also a major contributor to BVOC emissions, complicating its overall effects on environmental comfort. BVOC emissions in urban settings were often neglected due to the inadequate representation of urban landscapes [24]. By leveraging high-resolution land cover dataset (see Methods), we developed an urban BVOC emission inventory for metropolitan regions (Fig. S17). It is estimated that these metropolitan areas emit between 28.31 and 177.20 mol C km^−2^ h^−1^ of BVOCs (comprising isoprene, monoterpenes, and sesquiterpenes) from urban areas. Although these emissions are relatively low compared to those from natural forests, urban BVOCs exert a disproportionately large influence on O_3_ chemistry within cities (Fig. 4a), since they are spatially overlapped with dense urban NOx emission. Quantitatively, despite the dual positive effects in alleviating urban heat and O_3_ pollution meteorologically, BVOCs emitted from urban landscapes contribute to an increase in daytime O_3_ level by 0.67–3.31 μg m^−3^ in urban areas (Fig. S15b). The most significant increase in urban O_3_ levels due to greenery-boosted biogenic emissions is observed in the Tokyo-Pacific Coast UA, which is characterized by intensive urban BVOC emissions and heightened O_3_ sensitivity to VOCs.

Urban greenery influences O_3_ pollution through modifying urban climate or emitting substantial BVOCs. However, the extent to which such compound events may respond to urban greenery corresponds with the variations in O_3_ chemical regimes and urban vegetation coverage among regions. From a city-level perspective, analysis of H_2_O_2_/HNO_3_ from 198 cities reveals that O_3_ formation in most European and Asian cities is chemically sensitive to VOCs, that are characterized by sparse vegetation, intensive emissions and elevated NO_2_ (Fig. 4b and Fig. S18), whereas US cities fall into NOx-limited or mixed regimes. Regime-dependent responses of urban O_3_ pollution to greenery are further interpreted in Fig. 5, representing 20 cities suffering from severe compound heat-O_3_ pollution events. Cities along the Atlantic Coast exhibit a marked enhancement in O_3_ through both greenery-modified urban climate and biogenic emission, leading to an increase in daytime O_3_ levels by up to 7.6 μg m^−3^ during heatwaves. In the Tokyo-Pacific Coast UA with dense urban forest and intensive BVOC emission, the adverse effects of urban greenery as BVOC emitters on exacerbating O_3_ pollution often outweigh the benefits of its cooling effects in these cities, resulting in a substantial increase of daytime O_3_ up to 6.2 μg m^−3^. Whereas in other cities situated in Asia and Europe, urban O_3_ remains unchanged or shows minor increases, due to the competitive interactions between meteorological and biochemical effects by urban greenery. As anthropogenic VOC emissions are expected to decline under clean-energy transitions and air temperatures will continue to rise, it is anticipated that BVOC emissions from urban landscapes will play an increasingly prominent role in shaping O_3_ pollution.

**Figure 5.**
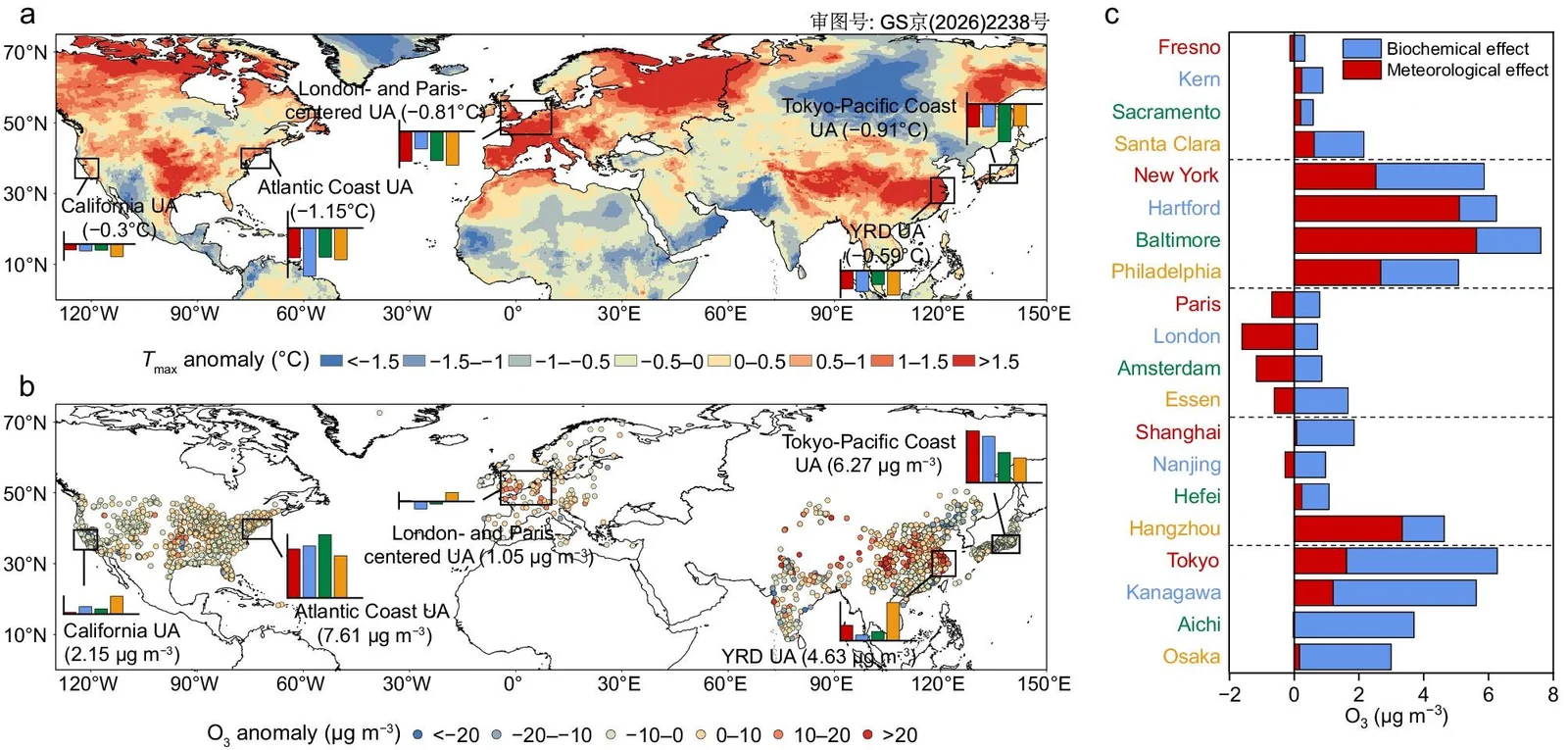
Effects of urban greenery on heat and O_3_ pollution during compound events in 20 typical Northern Hemisphere cities. (a) Cooling effect induced by urban greenery in the urban areas of 20 cities. *T*_max_ anomaly in the summer of 2022 relative to the 2014–2023 averages (shaded areas) is shown in panel (a). Maximal value of city-level cooling effect is given under each bar figure. (b) Greenery-induced changes of daytime surface O_3_ concentrations in the urban areas of 20 cities. Surface O_3_ anomaly in the summer of 2022 relative to the 2014–2023 averages (shaded areas) is shown in panel (b). Maximal O_3_ increase is given under each bar figure. (c) Greenery-induced changes of daytime surface O_3_ concentrations in the urban areas of 20 cities through meteorological (red bar) and biochemical (blue bar) mechanisms. Four cities from each metropolitan region are shown with the same color as those in panels (a, b).

## DISCUSSION

The Northern Hemisphere has recently experienced its warmest period on record, with increasing heat extremes in both the frequency and intensity at rates of 1.0 days decade^−1^ and 6.6°C decade^−1^ (*P* < 0.05). The simultaneous occurrence of elevated air temperatures and O_3_ levels has emerged as a principal urban challenge. Using an integrated urban climate–chemistry modeling framework with realistic representations of urban landscapes, we show that urban greenery provides a substantial cooling benefit, reducing *T*_max_ by 0.26 to 1.08°C across metropolitan areas. This cooling effect varies with the fraction of urban vegetation and the climate-dependent cooling efficiency. As illustrated in Fig. 4, the interactions among vegetation, urban meteorology, and O_3_ pollution are complex. Surface O_3_ typically decline in urban centers due to the nonlinearly weakened chemical production and altered airflow patterns induced by urban greenery. However, the increased BVOC emissions from urban vegetation outweigh the cooling-driven co-benefits, ultimately leading to a total increase in urban O_3_ up to 7.6 μg m^−3^ in typical cities during compound events.

We further evaluate the benefits of urban greenery in reducing population exposure to extreme high temperatures (*T*_max_ > 32°C), O_3_ pollution (MDA8 O_3_ > 120 μg m^−3^) and their co-occurrences (Fig. 6). Our analysis reveals a notable trade-off between the climatic and environmental impacts of urban greenery: while it reduces exposure to extreme heat and compound heat-O_3_ pollution events, benefiting between 9.51% and 43.19% of the population in metropolitan regions, it may inadvertently elevate exposure to O_3_ pollution alone. Notably, mitigation of compound heat-O_3_ pollution events, which pose a particularly severe public health risk, could benefit ∼7.62 million individuals.

**Figure 6.**
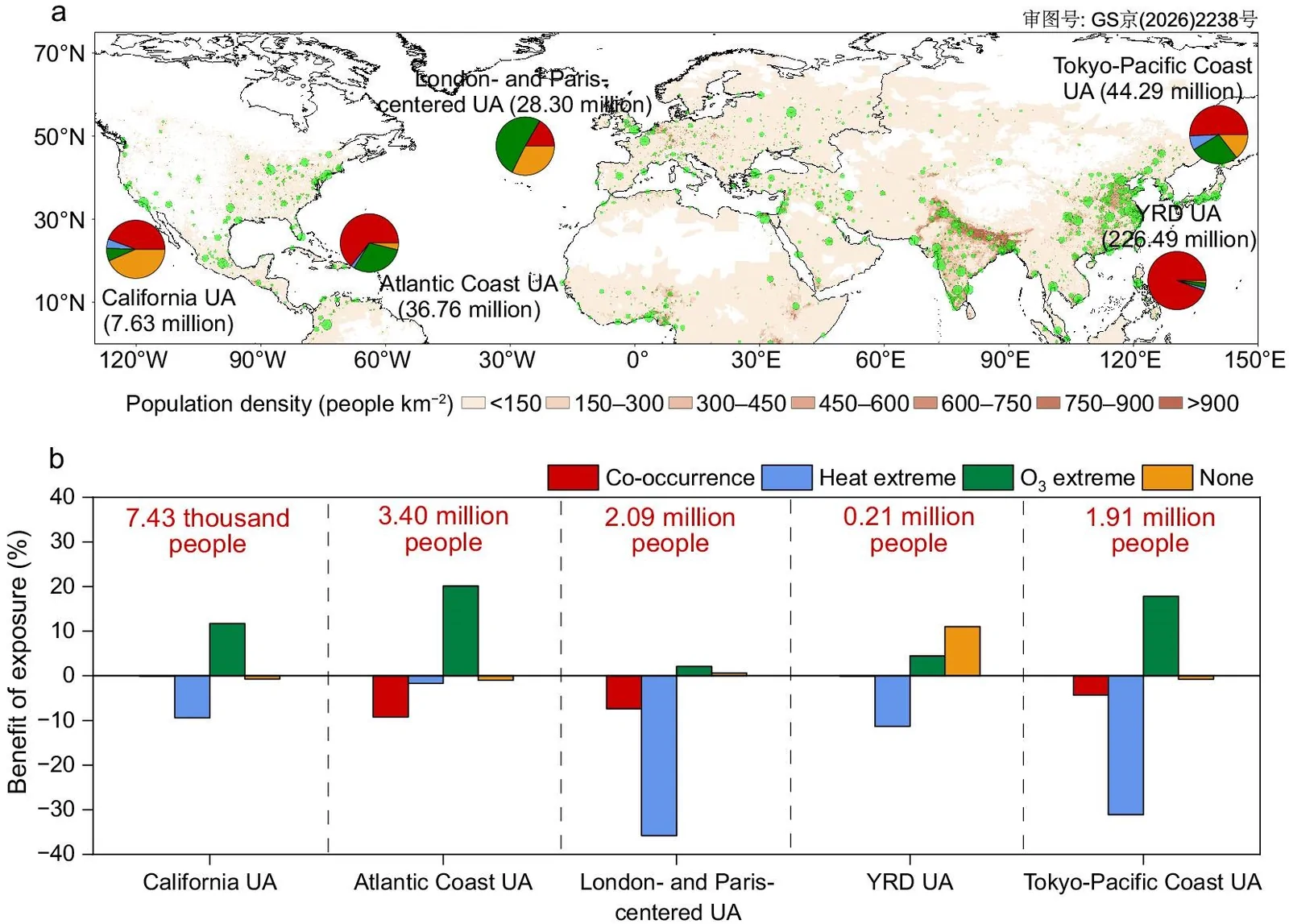
Benefits of population exposure to urban heat and O_3_ pollution due to urban greenery. (a) Population exposed to heat extremes (blue pie), O_3_ pollution (green pie), and their compound events (red pie) in the five metropolitan regions. Values given under each pie chart denote the total population exposed to compound event. Population density obtained from the Gridded Population of the World, version 4 (GPWv4; shaded areas), and cities with more than 300 thousand population obtained from the World Urbanization Prospects [1] (green dots) are shown in panel (a). (b) Benefits of population exposed to heat extremes, O_3_ pollution and their compound events due to urban greenery in the five metropolitan regions. Reduced population exposure to compound heat-O_3_ pollution event is given in panel (b).

The escalating pressures of global warming have intensified the urgency for urban greening initiatives. In 2015, 196 nations ratified Paris Agreement, committing to limit the increase in global average air temperature from pre-industrial levels to below 1.5°C by the end of 21st century. In response, numerous large-scale tree-planting and greening programs have been launched to enhance urban biodiversity and climate resilience, including China’s National Ecological Garden City program [29] and EU’s 3 Billion Trees Initiative [28]. While both this and previous works reveal temperature reductions owing to the presence of greenery, the magnitude of cooling varies substantially [39]. For instance, a meta-analysis covering tropical and temperate climates indicates air temperature cooling of 0.94°C [14], whereas another synthesis of 89 studies suggests cooling of 1.5°C–3.5°C [40], much higher than the estimates in this study. Beyond greenery fraction and local climate discussed in this study, the cooling efficiency of urban vegetation is strongly shaped by urban morphology, tree species composition, green space configuration, and vegetation structure, exerting complex and nonlinear cooling effects in cities [16,41,42]. Many previous studies focus on localized park-scale cooling or specific green infrastructure types, which can produce strong microclimatic cooling. Whereas, our estimate represents urban-region-scale mean response, in which urban green spaces are parameterized in a simplified manner to calculate surface energy balance via area-weighted averaging of impervious and vegetated surfaces (see Methods). Therefore, fine-scale spatial configurations of green spaces and three-dimensional canopy characteristics, such as leaf area index (LAI), canopy height, and tree-species differences, are not explicitly resolved. A more comprehensive evaluation of these interacting factors based on urban microclimate models that incorporate detailed vegetation canopy structures is essential while supporting broader environmental and societal goals. To address these issues, some sophisticated three-dimensional urban microclimate models, such as ENVI-met [43] and BEP-Tree [44], have integrated detailed vegetation canopy structures, cooling mechanisms, and the interactions with urban form, thereby enabling higher-resolution simulations of urban thermal environments.

Our research suggests that while urban greening is a nature-based strategy for urban heat mitigation, it may inadvertently exacerbate O_3_ pollution due to complex interactions between vegetation and climate. Nonetheless, considerable uncertainties persist in urban BVOC emission estimation, arising from the representation of vegetation fraction, LAI, and tree species composition [22,23], species-specific emission potentials [45,46], and local environment-dependent functions [47]. Bao, Zhou [22] and Zhang, Ran [23] have demonstrated that coarse-resolution plant functional type data may substantially underestimate BVOC emissions (∼20%) compared with tree-species-based inventories, especially in urban areas where tree species composition is highly heterogeneous. In addition, BVOC emission factors (EFs) vary markedly across vegetation types, species, and regions, sometimes by nearly an order of magnitude [45,46], highlighting the need for locally measured EFs. Furthermore, many BVOC emission algorithms are derived from experiments under normal environmental conditions, whereas their applicability under heat extremes, drought stress, and urban canopy microclimates remains uncertain [47].

Notably, several complex feedback mechanisms are not fully addressed in this study. For example, increased BVOC emissions from urban greenery can promote the formation of secondary organic aerosols, potentially posing extra cooling effect. Nevertheless, recent modeling studies suggested that such aerosol-related thermal feedback is generally weaker than direct cooling of urban greenery through transpiration and shading [48]. Additionally, urban vegetation removes O_3_ via dry deposition and counteracts the adverse effect of BVOC emissions. This process is governed by plant physiological characteristics (e.g. tree species, LAI, and leaf roughness). Dry deposition in WRF/Chem is represented using the empirical resistance-in-series approach proposed by Wesely [49] (see Methods). Stomatal resistance is parameterized based on the multiplicative approach of Jarvis [50], in which a high stomatal resistance is prescribed for urban vegetation, implying the dominance of non-stomatal uptake in urban areas. Accordingly, model configurations here cannot isolate the contribution of stomatal deposition by urban vegetation to air pollution. Hence, future work is still needed to integrate process-based stomatal deposition, secondary aerosol feedbacks, and fine-scale vegetation canopy structures into urban models. Such advances are essential for mechanistically resolving how urban greening modifies heat and O_3_ exposure under extreme climate conditions, and for guiding climate-adaptive greening strategies that maximize cooling benefits while minimizing unintended air-quality trade-offs.

## MATERIALS AND METHODS

The detailed materials and methods are available as Supplementary Information.

## Supplementary Material

nwag494_Supplemental_File

## Data Availability

The ERA5 reanalysis data are available at www.ecmwf.int. Land-based weather data are archived at the Integrated Surface Database (www.ncei.noaa.gov/pub/data). Surface O_3_ and isoprene concentrations are downloaded from the US Environmental Protection Agency (https://aqs.epa.gov/aqsweb/airdata), European Monitoring and Evaluation Programme (EMEP) (https://ebas-data.nilu.no), China’s Ministry of Ecology and Environment (https://quotsoft.net/air), India’s Central Pollution Control Board (https://cpcb.nic.in/), and Japan’s National Institute for Environmental Studies (https://tenbou.nies.go.jp/download/). GPWv4 population data are archived at NASA’s Socioeconomic Data and Applications Center (SEDAC; https://beta.sedac.ciesin.columbia.edu/data). The FROM-GCL10 dataset is archived at https://data-starcloud.pcl.ac.zn/zh. WRF/Chem model code is available at https://github.com/wrf-model/WRF.
