# Supplementary material for: Unraveling the potential of urban greenery for extreme heat and ozone mitigation in Northern Hemisphere cities
Source: Natl Sci Rev. 2026 Aug 17;13(17):nwag494. doi: 10.1093/nsr/nwag494 (PMC13639184; doi:10.1093/nsr/nwag494)
Supplement: Supplementary file 1 [file nwag494_supplemental_file.docx]

**Supplementary information for**

**Unraveling the potential of urban greenery for extreme heat and ozone mitigation in Northern Hemisphere cities**

Mengmeng Li^1,2,3^, Yuting Lu^2^, Xin Huang^1,2,3,*^, Hui Zhang^2^, Zilin Wang^1,2^, Yue Qin^4^, Jun Ge^2^, Xueyu Zhou^2^, Wei Wang^5^, Wenjie Wang^5^, Nan Wang^6^, Tijian Wang^2^, Minghuai Wang^1,2,3^, Haishan Chen^7^, Aijun Ding^2,5^

^1^State Key Laboratory of Severe Weather Meteorological Science and Technology, Nanjing University, Nanjing 210023, China

^2^School of Atmospheric Sciences, Nanjing University, Nanjing 210023, China

^3^Frontiers Science Center for Critical Earth Material Cycling, Nanjing 210023, China

^4^College of Environmental Sciences and Engineering, Peking University, Beijing 100871, China

^5^Nanjing-Helsinki Institute in Atmospheric and Earth System Sciences, Nanjing University, Suzhou 215163, China

^6^College of Carbon Neutrality Future Technology, Sichuan University, Chengdu 610207, China

^7^State Key Laboratory of Climate System Prediction and Risk Management, Nanjing University of Information Science & Technology, Nanjing 210044, China

^*^Correspondence: xinhuang[@](mailto:songyu@pku.edu.cn)nju.edu.cn

**This file includes:**

Materials and methods

References cited in the materials and methods

Table S1

Figures S1 to S18

**Materials and methods**

**Data**

*Global reanalysis and land-based weather data.* Here, we utilize the fifth-generation European Centre for Medium-range Weather Forecasts (ECMWF) reanalysis (ERA5) data to characterize extreme heat events in the Northern Hemisphere [1]. This dataset has a spatial resolution of 0.25° by 0.25°, and a temporal resolution of 1 hour. Hourly 2-m air temperature data for June to August from 1980 to 2023 are utilized to calculate the daily maximum air temperature (*T*_max_) and to analyze the spatio-temporal trends of extreme heat events. Additionally, hourly or 3-hourly 2-m air temperature observations from land-based weather stations across the Northern Hemisphere, which are archived at the Integrated Surface Database of National Oceanic and Atmospheric Administration (NOAA), are used to evaluate the model’s meteorological performance.

*Surface ozone (O_3_) concentrations in the Northern Hemisphere.* Hourly surface O_3_ concentrations for June–August in the past decade (2014–2023) are collected from major countries in the Northern Hemisphere, including China, India, Japan, Europe, and the United States (US). Specifically, surface O_3_ concentrations at approximately 3,000 monitoring stations across the US are provided by the Environmental Protection Agency (EPA) at <https://aqs.epa.gov/aqsweb>. Surface O_3_ concentrations at more than 1,500 monitoring stations across China are downloaded from https://quotsoft.net/air, a mirror of the Ministry of Ecology and Environment (MEE). Surface O_3_ concentrations at 232 monitoring stations across western European are downloaded from the European Monitoring and Evaluation Programme (EMEP) at <https://ebas-data.nilu.no>. Surface O_3_ concentrations across India at 269 monitoring stations are obtained from the Central Pollution Control Board at https://cpcb.nic.in/. Surface O_3_ concentrations at more than 1,000 monitoring stations across Japan are downloaded from the National Institute for Environmental Studies (NIES) at <https://tenbou.nies.go.jp/download/>. The observed O_3_ concentrations are detrended and deseasonalized to remove the long-term trends and seasonal cycles, so as to facilitate comparison in different regions or time periods [2].

*Ambient isoprene concentrations.* Ambient isoprene concentrations were continuously measured at the Essex site located in Baltimore (39.31°N, –76.47°W) and North Main Street site located in Los Angeles (34.07°N, –118.23°W), which were operated by the US EPA. Additionally, hourly isoprene data were measured at Chilbolton site (51.15°N, –1.43°W) that is located in the Great Britain and operated by EMEP. Real-time isoprene concentrations were also measured at the Station for Observing Regional Processes of Earth System (SORPES; 32.12°N, 118.95°E) in Nanjing City, China since 2011 [3]. These observatory sites are all located in the city centers or suburbs of study areas.

*Satellite data.* Remote sensing enables the complete spatial monitoring of vegetation greenness in urban and its adjacent regions. This research employs the monthly, 1 km resolution Normalized Difference Vegetation Index (NDVI) product between 2010 and 2022 to assess urban vegetation greenness, which is derived from the visible and near-infrared data collected by Moderate Resolution Imaging Spectroradiometer (MODIS) [4]. Meanwhile, satellite-derived land surface temperature (LST) is retrieved from the Collection 6 MODIS LST products (MOD11A2 and MYD11A2), which are generated using the generalized split-window algorithm [5], with an 8-day temporal resolution and a 1 km spatial resolution. The products collectively provide LST observations four times daily (10:00 and 22:00 for the Terra satellite, and 13:30 and 01:30 for the Aqua satellite). Petitcolin and Vermote [6] have determined that the accuracy of MODIS LST is within ±1 °C. To reduce the effects of cloud contamination, only LST observations with a retrieval error less than 3.0 °C are considered [7]. All datasets are resampled to a 1 km resolution using the nearest neighbor method.

**Definition of compound heat-ozone event**

So far, there is no universally accepted definition for extreme heat events. We define an extreme heat event as a day when *T*_max_ surpasses 32 °C, a threshold recommended by World Meteorological Organization (WMO). The occurrence frequency of extreme heat events is quantified as the total number of days that exceed this *T*_max_ threshold. The cumulative intensity of extreme heat events refers to the total excess heat above this temperature threshold. Analyses with different *T*_max_ thresholds of heat extremes show large differences in spatial patterns and quantities (see Results). Since the interactions between temperature and O_3_ concentration depend on absolute temperature magnitudes, using WMO’s fixed temperature threshold could better characterizes the temperature dependence of O_3_.

A compound heat-O_3_ pollution event is defined as a day when both *T*_max_ exceeds 32 °C and maximum daily 8-hour average (MDA8) O_3_ concentration exceeds the World Health Organization (WHO)’s Interim Target II of 120 μg m^-3^. Analyses with different definitions for compound heat-O_3_ pollution events show that frequency of compound events differs in magnitude but exhibits consistent spatial patterns, that concentrated in the densely populated and highly polluted urban agglomerations (UA; see Results).

Population exposures to extreme heat (*T*_max_>32 °C), O_3_ pollution (MDA8 O_3_>120 μg m^−3^) and their co-occurrences are quantified using meteorology-chemistry model outputs and GPWv4 global population density data archived at NASA’s Socioeconomic Data and Applications Center (SEDAC; <https://beta.sedac.ciesin.columbia.edu/data>). Exposure is defined as the number of people affected by such extreme events.

**WRF/Chem-SLUCM model configurations**

The Weather Research and Forecasting/Chemistry (WRF/Chem) model version 4.3 is employed to simulate the interactions between urban meteorology and atmospheric chemistry during compound heat-O_3_ pollution events. Two-nested modeling domains are configured focusing on these metropolitan regions, with a horizontal resolution of 18 km and 6 km, respectively. Vertically, the model setups 24 eta levels extending from the surface to 100 hPa. The initial and boundary meteorological conditions are derived from 6-hour, 0.25°×0.25° ECMWF reanalysis data. Anthropogenic emissions adopt the 0.1°×0.1° resolution Emissions [Database](https://so.csdn.net/so/search?q=Database&spm=1001.2101.3001.7020) for Global Atmospheric Research (EDGAR; https://edgar.jrc.ec.europa.eu/). Biogenic volatile organic compound (BVOC) emissions are calculated online using the Model of Emissions of Gases and Aerosols from Nature (MEGAN) version 2.04 [8]. Gas-phase chemistry is modelled using the Carbon Bond Mechanism [9]. Physical options include the Yonsei University boundary layer scheme [10], the Goddard short-wave radiation scheme [11], and the Rapid Radiative Transfer Model (RRTM) long-wave radiation scheme [12].

Urban canopy processes are parameterized using the Single-Layer Urban Canopy Model (SLUCM) [13] coupled with Noah land surface model (LSM) [14], which has been validated to reliably reproduce the urban meteorology and hydrological cycle [15]. In the coupled Noah/SLUCM model, urban surface energy balance is expressed as **Eq. S1**. Heat flux released into the atmosphere over an urban grid is calculated as the area-weighted average of contributions from impervious surface simulated by the SLUCM and vegetated surface represented by the Noah LSM. SLUCM accounts for the three-dimensional structures of urban surfaces, including street canyons, building shadowing, radiation reflection and trapping within the urban canopy, as well as a multi-layer heat equation applicable to the roofs, walls, and roads. Improved urban hydrological process schemes, including anthropogenic latent heat emission, urban oasis effect, irrigation of urban landscape, and a refined evaporation mechanism for impervious urban surface, have been incorporated into the SLUCM [16].

$Q^{*}+Q_{AH}=Q_{H}+Q_{E}+Q_{S}$ (Eq. S1)

$$Q_{E}=f_{urb}(Q_{E,urb}+Q_{ALH})+f_{veg}Q_{E,veg}$$

where *Q^*^* is the net radiation flux; *Q_AH_* is the anthropogenic heat flux; *Q_H_* is the sensible heat flux; *Q_S_* is the ground heat storage; *Q_E_* is the latent heat flux that can be expressed as an area-weighted average of contributions from impervious urban surfaces (*Q_E, urb_*) and vegetated urban surfaces (*Q_E, veg_*); *f_urb_* and *f_veg_* are the fractions of impervious and vegetated urban surfaces, respectively; *Q_ALH_* is the anthropogenic latent heat flux.

Specifically, *Q_E, veg_* is derived as a sum of soil evaporation (*E_S_*), plant transpiration (*E_TT_*; **Eq. S2**), canopy interception evaporation (*E_C_*), and snow sublimation (*E_SNOW_*).

$E_{TT}=E_{pot}B_{c}[1-(\frac{W_{c}}{S})^{n}]$ (Eq. S2)

where *E_pot_* is the potential evaporation calculated via **Penman-Monteith equation [17, 18], that is dependent on ambient weather conditions such as net radiation, temperature, and humidity**; *B_c_* is the canopy resistance function that characterizes stomatal resistance to water vapor transfer; *W_c_* represents the canopy intercepted water storage; *S* denotes the maximum canopy water capacity; *n* is an empirical coefficient set to 0.5.

**Experimental designs**

In this study, 500 m resolution MODIS land cover product (MCD12Q1) is incorporated into WRF to describe urban distributions [19]. Gong, Liu [20] have demonstrated that MODIS urban land classification exhibited a high accuracy of 93% at both pixel and city levels. Urban areas are then categorized into three urban types—low-intensity residential area (referred to as “suburban region”), high-intensity residential area, and commercial center (together referred to as “urban center”; **Fig. S6**). This classification is based on the GPWv4 global population density data. Look-up table is applied to allocate urban canopy parameters such as building height and width, street width, and anthropogenic heat to the three urban types [13]. Meanwhile, an urban grid is divided into impervious and vegetated fractions, based on which total urban surface heat flux can be quantified (**Eq. S1**). Here, the fractions of impervious and vegetated urban surfaces are estimated from the 10-m Finer Resolution Observation and Monitoring of Global Land Cover (FROM-GLC10) dataset [20]. This dataset is generated from the publicly available Landsat Thematic Mapper imagery and adopts a 10-type land cover classification scheme (i.e., cropland, forest, grassland, shrubland, wetland, water, tundra, impervious surface, bareland, and snow). It indicates that the vegetated fraction of urban land varies from less than 20% in urban centers to nearly 50% in the suburban regions (**Fig. S6**).

To examine the benefits of urban greenery in mitigating urban heat extremes, a series of WRF-SLUCM model scenarios are devised (**Table S1**). Specifically, in the baseline *URB* scenario, the fractions of vegetated urban land are set as zero over the innermost domain. Conversely, the *URG* scenario employs high-resolution FROM-GLC10 dataset to represent the fractions of urban grids occupied by natural vegetation. All the WRF- SLUCM simulation scenarios are performed for the period from June to August 2022, representing an exceptionally hot summer over the past decade.

Furthermore, to explore the co-benefits of urban greenery in mitigating compound heat-O_3_ pollution, another series of WRF/Chem-SLUCM model scenarios (**Table S1**) are devised for typical prolonged heat-O_3_ pollution events in the summer of 2022 (**Fig. S4**). In the *URB* and *URG* scenarios, we assume that urban BVOC emissions do not occur. Comparisons between *URG* and *URB* represent the impacts of greenery-altered urban climate on urban O_3_ pollution (referred to as “meteorological effect”). To elucidate the environmental impacts of urban greenery through increased urban BVOC emissions, the high-resolution FROM-GLC10 dataset is then utilized to estimate BVOC emissions from urban landscapes, referred to as the *U-BVOC* scenario. The FROM-GLC10 dataset includes four vegetation types (i.e., forest, cropland, grassland, and shrub), which are then mapped to the four PFTs in MEGAN (i.e., broadleaf forest, coniferous forest, shrub, and grassland/cropland) as suggested by Ma et al. [21]. Comparisons between the *U-BVOC* and *URG* scenarios characterize the influence of urban greenery on O_3_ pollution resulting from increased urban BVOC emissions (referred to as “biochemical effect”).

Notably, dry deposition in WRF/Chem is simulated using the empirical resistance-in-series approach proposed by Wesely [22]. Deposition velocity is determined by aerodynamic resistance, quasi-laminar boundary-layer resistance, and surface/canopy resistance. Stomatal resistance is parameterized based on the multiplicative approach of Jarvis [23] (**Eq. S3**), failing to explicitly account for the effects of urban vegetation phenology on stomatal uptake. A relatively high canopy stomatal resistance is prescribed for urban vegetation, implying the dominance of non-stomatal uptake in urban areas. Accordingly, model configurations in this study cannot isolate the contribution of stomatal deposition by urban vegetation to air pollution levels.

$R_{\text{s}}=r_{\text{m}}\times(1+(200\times(G+0.1)^{-1})^{2})\times(400\times(T\times(40-T))^{-1})\times\frac{D_{\text{H}_{2}\text{O}}}{D_{\text{O}_{3}}}$ (Eq. S3)

where *R*_s_ is the canopy stomatal resistance; *r*_m_ is the minimum stomatal resistance which is predefined via a look-up table dependent on vegetation type and season; *G* is solar radiation; *T* is air temperature; *D*_H2O_ and *D*_O3_ represent the [molecular](javascript:;) [diffusivity](javascript:;) for vapor and O_3_, respectively.

**References**

1.	Hersbach H, Bell B, Berrisford P *et al.* The ERA5 global reanalysis. *Q J Roy Meteor Soc* 2020; **146**: 1999–2049.

2.	Santiago JV, Hata H, Martinez-Noriega EJ *et al.* Ozone trends and their sensitivity in global megacities under the warming climate. *Nat Commun* 2024; **15**: 10236.

3.	Ding AJ, Fu CB, Yang XQ *et al.* Ozone and fine particle in the western Yangtze River Delta: an overview of 1 yr data at the SORPES station. *Atmos Chem Phys* 2013; **13**: 5813–5830.

4.	Justice CO, Townshend JRG, Vermote EF *et al.* An overview of MODIS Land data processing and product status. *Remote Sens Environ* 2002; **83**: 3–15.

5.	Wan Z, Zhang Y, Zhang Q *et al.* Quality assessment and validation of the MODIS global land surface temperature. *Int J Remote Sens* 2004; **25**: 261–274.

6.	Petitcolin F, Vermote E. Land surface reflectance, emissivity and temperature from MODIS middle and thermal infrared data. *Remote Sens Environ* 2002; **83**: 112–134.

7.	Venter ZS, Chakraborty T, Lee XH. Crowdsourced air temperatures contrast satellite measures of the urban heat island and its mechanisms. *Sci Adv* 2021; doi: 10.1126/sciadv.abb956.

8.	Guenther A, Karl T, Harley P *et al.* Estimates of global terrestrial isoprene emissions using MEGAN (Model of Emissions of Gases and Aerosols from Nature). *Atmos Chem Phys* 2006; **6**: 3181–3210.

9.	Zaveri RA, Peters LK. A new lumped structure photochemical mechanism for large-scale applications. *J Geophys Res-Atmos* 1999; **104**: 30387–30415.

10.	Noh Y, Cheon WG, Hong SY *et al.* Improvement of the K-profile model for the planetary boundary layer based on large eddy simulation data. *Bound-Lay Meteorol* 2003; **107**: 401–427.

11.	Chou MD, Suarez MJ. A shortwave radiation parameterization for atmospheric studies. *NASA/TM-104606*. 1999, 40 pages.

12.	Gallus WA, Bresch JF. Comparison of impacts of WRF dynamic core, physics package, and initial conditions on warm season rainfall forecasts. *Mon Weather Rev* 2006; **134**: 2632–2641.

13.	Kusaka H, Kondo H, Kikegawa Y *et al.* A simple single-layer urban canopy model for atmospheric models: Comparison with multi-layer and slab models. *Bound-Lay Meteorol* 2001; **101**: 329–358.

14.	Chen F, Dudhia J. Coupling an advanced land surface-hydrology model with the Penn State-NCAR MM5 modeling system. Part I: Model implementation and sensitivity. *Mon Weather Rev* 2001; **129**: 569–585.

15.	Holt T, Pullen J. Urban canopy modeling of the New York City metropolitan area: A comparison and validation of single- and multilayer parameterizations. *Mon Weather Rev* 2007; **135**: 1906–1930.

16.	Yang JC, Wang ZH, Chen F *et al.* Enhancing hydrologic modelling in the coupled Weather Research and Forecasting-urban modelling system. *Bound-Lay Meteorol* 2015; **155**: 369–369.

17.	Penman HL. Natural evaporation from open water, bare soil and grass. *Proc R Soc Lon Ser-A* 1948; **193**: 120–125.

18.	Monteith JL. Evaporation and environment. *Symp Soc Exp Biol* 1965; **19**: 205–234.

19.	Li MM, Wang TJ, Xie M *et al.* Improved meteorology and ozone air quality simulations using MODIS land surface parameters in the Yangtze River Delta urban cluster, China. *J Geophys Res-Atmos* 2017; **122**: 3116–3140.

20.	Gong P, Liu H, Zhang MN *et al.* Stable classification with limited sample: transferring a 30-m resolution sample set collected in 2015 to mapping 10-m resolution global land cover in 2017. *Sci Bull* 2019; **64**: 370–373.

21.	Ma MC, Gao Y, Ding AJ *et al.* Development and assessment of a high-resolution biogenic emission inventory from urban green spaces in China. *Environ Sci Technol* 2022; **56**: 175–184.

22.	Wesely ML. Parameterization of surface resistances to gaseous dry deposition in regional-scale mumerical-models. *Atmos Environ* 1989; **23**: 1293–1304.

23.	Jarvis PG. The interpretation of the variations in leaf water potential and stomatal conductance found in canopies in the field. *Philos Trans R Soc B* 1976; **273**: 593–610.

**Table S1**. WRF/Chem-SLUCM experimental designs in this study

| **Scenarios** | **Vegetated fractions of urban grid in SLUCM** | **Urban BVOC emissions in MEGAN** |
| --- | --- | --- |
| *URB* | None | None |
| *URG* | FROM-GLC10 | None |
| *U-BVOC* | FROM-GLC10 | FROM-GLC10 |

**Figure S1.** Spatial distributions of summertime heat extremes during 2014–2023 based on different thresholds: (a) WMO’s fixed threshold of 32 °C; (b) CMA’s fixed threshold of 35 °C; and (c) 90^th^ percentiles of local *T*_max_ distributions as shown in panel (d).

**Figure S2.** (a) Summer-average MDA8 O_3_ concentrations between 2014 and 2023. (b) Correlation coefficients (*R*) between the deseasonalized and detrended MDA8 O_3_ concentrations with *T*_max_. (c) Elevated MDA8 O_3_ concentrations during heat extremes compared to normal days.

**Figure S3.** Frequency of compound heat-O_3_ extremes during 2014–2023 based on different *T*_max_ and O_3_ thresholds: (a–c) MDA8 O_3_ concentration exceeds WHO’s Interim Target II of 120 μg m^-3^, while *T*_max_ exceeds WMO’s fixed threshold of 32 °C (a), CMA’s fixed threshold of 35 °C (b), and 90^th^ percentiles of local *T*_max_ distributions (c). (d–f) MDA8 O_3_ concentration exceeds China’s Ambient Air Quality Standard Grade II of 160 μg m^-3^, while *T*_max_ exceeds WMO’s fixed threshold of 32 °C (d), CMA’s fixed threshold of 35 °C (e), and 90^th^ percentiles of local *T*_max_ distributions (f).

**Figure S4.** Daily timeseries of *T*_max_ and MDA8 O_3_ concentrations during 2022 summer compared to the 2014–2023 averages in five urban agglomerations of the Northern Hemisphere. Grey shaded areas denote several instances of compound heat-O_3_ events.

**Figure S5.** (a) Biannual trends of summer-average MDIS NDVI in the urban centers of five urban agglomerations from 2010 to 2022. Relative changes of NDVI are given under each bar chart. Shaded area represents distribution of summer-average NDVI. (b) Summer-average NDVI in the urban centers (blue bars) and urban areas (red bars) of five urban agglomerations.

**Figure S6.** Urban types (upper panels) and impervious urban fractions (bottom panels) in the five metropolitan regions.

**Figure S7.** (a) Spatial distributions of summer-average 2-m air temperature (T2) from the WRF-SLUCM urban climate simulations and site observations. (b) Daily timeseries of site-average 2-m air temperature from the WRF-SLUCM urban climate simulations and observations.

**Figure S8.** Changes in surface latent heat (upper panels) and sensible heat (bottom panels) due to urban greenery during 2022 summer from WRF-SLUCM urban climate simulations. The average values calculated over urban areas are given in each panel.

**Figure S9.** Relationship between the summer-average MODIS LST and NDVI in five capital cities within these metropolitan regions during 2022 summer. Correlation coefficients and slopes between LST and urban NDVI are given in each panel.

**Figure S10.** Cooling effects with varying temperatures in (a) the California UA, (b) the Atlantic Coast UA, (c) the London- and Paris-centered UA, (d) the YRD UA, and (e) the Tokyo-Pacific Coast UA. Box charts denote the 25^th^ and 75^th^ percentiles of values.

**Figure S11.** (a) Spatial distribution comparisons of surface MDA8 O_3_ concentrations, (b) scatter plot comparisons of station-average hourly O_3_ concentrations, and (c) time-series comparisons of station-average hourly O_3_ concentrations during compound heat-O_3_ events from the *U-BVOC* model scenarios against ground observations.

**Figure S12.** Comparisons of the simulated isoprene concentrations from the *U-BVOC* model scenarios against in-situ observations at four observational sites: (a) North Main Street site in Los Angeles, (b) Essex site in Baltimore, (c) Chilbolton site in the Great Britain, and (d) SORPES site in Nanjing.

**Figure S13.** Effects of urban greenery on urban boundary layer height (upper panels) and daytime surface NO_2_ concentrations (bottom panels) during compound events. The average values calculated over urban areas are given in each panel.

**Figure S14.** Daytime H_2_O_2_/HNO_3_ ratios indicating the O_3_ photochemical sensitivity regimes in (a) the California UA, (b) the Atlantic Coast UA, (c) the London- and Paris-centered UA, (d) the YRD UA, and (e) the Tokyo-Pacific Coast UA.

**Figure S15.** Effects of urban greenery on daytime O_3_ concentrations through the (a) meteorological effect, (b) biochemical effect and (c) their combined effect during compound events. The average values calculated over urban areas are given in each panel.

**Figure S16.** Changes in daytime surface O_3_ chemical production due to (a) greenery-induced meteorological changes and (b) urban BVOC emissions in the YRD and Atlantic Coast **urban agglomerations**. (c) Greenery-induced changes in wind fields and advection contribution in the YRD and Atlantic Coast **urban agglomerations**.

**Figure S17.** Estimated BVOC emissions in (a) the California UA, (b) the Atlantic Coast UA, (c) the London- and Paris-centered UA, (d) the YRD UA, and (e) the Tokyo-Pacific Coast UA. Red and green shaded colors represent BVOC emissions from urban and rural regions, respectively. Averages values for urban (red texts) and rural (green texts) BVOC emissions are given in each panel.

**Figure S18.** Daytime surface NO_2_ concentration (a) and H_2_O_2_/HNO_3_ ratio (b) in the urban areas of 198 cities within these five metropolitan regions. *N* denotes the number of cities in each metropolitan region. Box charts denote the 25^th^ and 75^th^ percentiles of values across each metropolitan region.
